# The Actor in 4 dimensions: A relevant methodology to analyze local environmental governance and inform Ostrom’s social-ecological systems framework

**DOI:** 10.1016/j.mex.2019.07.025

**Published:** 2019-07-31

**Authors:** Raphaëlle Dancette, Léa Sebastien

**Affiliations:** a Université du Québec à Rimouski (UQAR), 310 allée des Ursulines, Rimouski, Québec, G5L 3A1, Canada; b Toulouse II University, Geode CNRS Research Center, 5, Antonio Machado Street, 31000 Toulouse, France

**Keywords:** The Actor in 4 dimensions and its territorial footprint, Actor in 4 dimensions (A4D), Social-ecological systems framework, Perceptions, Environmental governance, Participation, Actors

## Abstract

This paper presents the Actor in 4 dimensions (A4D) model as a complementary tool to the Social-ecological systems framework (SESF) in order to better integrate individual and groups’ representations into local environmental governance analysis. As the A4D is based on actors’ representations of their social-ecological system (SES) and of its governance, it mainly informs the *Actors* subsystem of the SESF, even if it can also give useful insights for other framework’s sub-systems. We define the SESF *actor*’s sub-tiers and the corresponding A4D indicators and highlight the complementarity between both approaches in order to operationalize the SESF. This parallel is exemplified by the case of Maio island (a small-scale fishing community in Cape Verde). Our comparison also highlights other assets of the A4D methodology for the advancement of environmental governance’s study.

• The A4D allows actors' participation and discussion on the SES and analyses common and divergent discourses and values between actors.
• The A4D points to power relations by integrating *strong, weak* and *absent* actors in its analysis.
• By highlighting subjective and reflexive elements, the A4D complements the SESF in their common attempt to analyze SES.

## Graphical abstract

**Figure:**
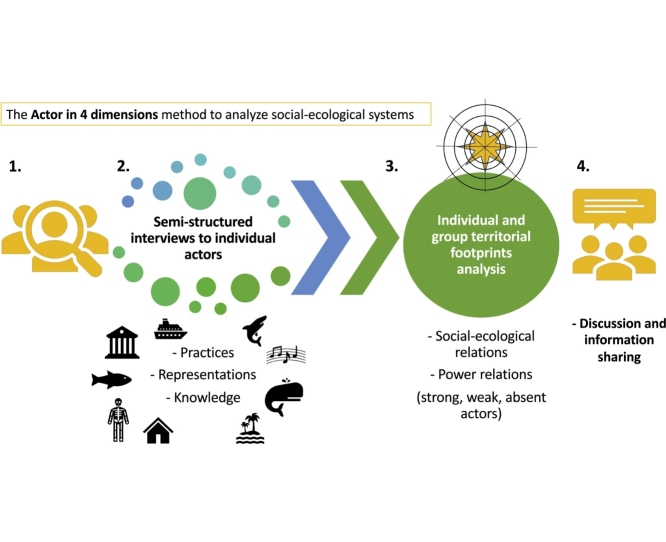

**Specifications Table**

**Table:** 

Subject Area:	Environmental Science
More specific subject area:	Local environmental governance
Method name:	The Actor in 4 dimensions and its territorial footprint
Name and reference of original method:	*L'Acteur en 4 Dimensions*; Sébastien L. & Paran F. (2006)
Resource availability:	L’Acteur en 4 Dimensions pour une exploration sociale et patrimoniale du jeu d’acteurs territorial" + "Pour un diagnostic socio-environnemental sur un territoire: l'Acteur en 4 Dimensions (A4D)" + "Le territoire, un système socio-patrimonial décrypté par le modèle de l’Acteur en 4 Dimensions" (Léa Sébastien, ResearchGate)

## Method details

The Actor in 4 dimensions (A4D) is a relevant and useful tool to complete the Social-ecological systems framework (SESF) through the inclusion of individual and groups' social representations into studies on local governance systems [[1], [2], [3]]. As a field tool (with interview guides and precise questions as exemplified in Annex 3), the A4D model proposes a specific method to study mental models, which can help operationalizing the SESF. The A4D adds to the SESF an approach to study individual perceptions and motivations [2,4,5], and meets the need to allow actors' participation and discussions on social-ecological systems (SES) [2,6].

A complementary use of the A4D and the SESF allows both a systemic and a strategic analysis of SES. First, social-ecological systems and their framework are defined [7,8] and their most common uses, described. Then, the A4D is detailed (specificities and assets) and the territorial footprint, presented. We show the relevance of this method to inform the SES framework by linking the A4D indicators with the SESF actors' tiers. *Authors*' tiers^1^ and indicators definitions are used, and links are validated by a specific case study from a small-scale fishing community in Maio, Cape Verde.

## The social-ecological system framework (SESF)

**Fig. 1:**
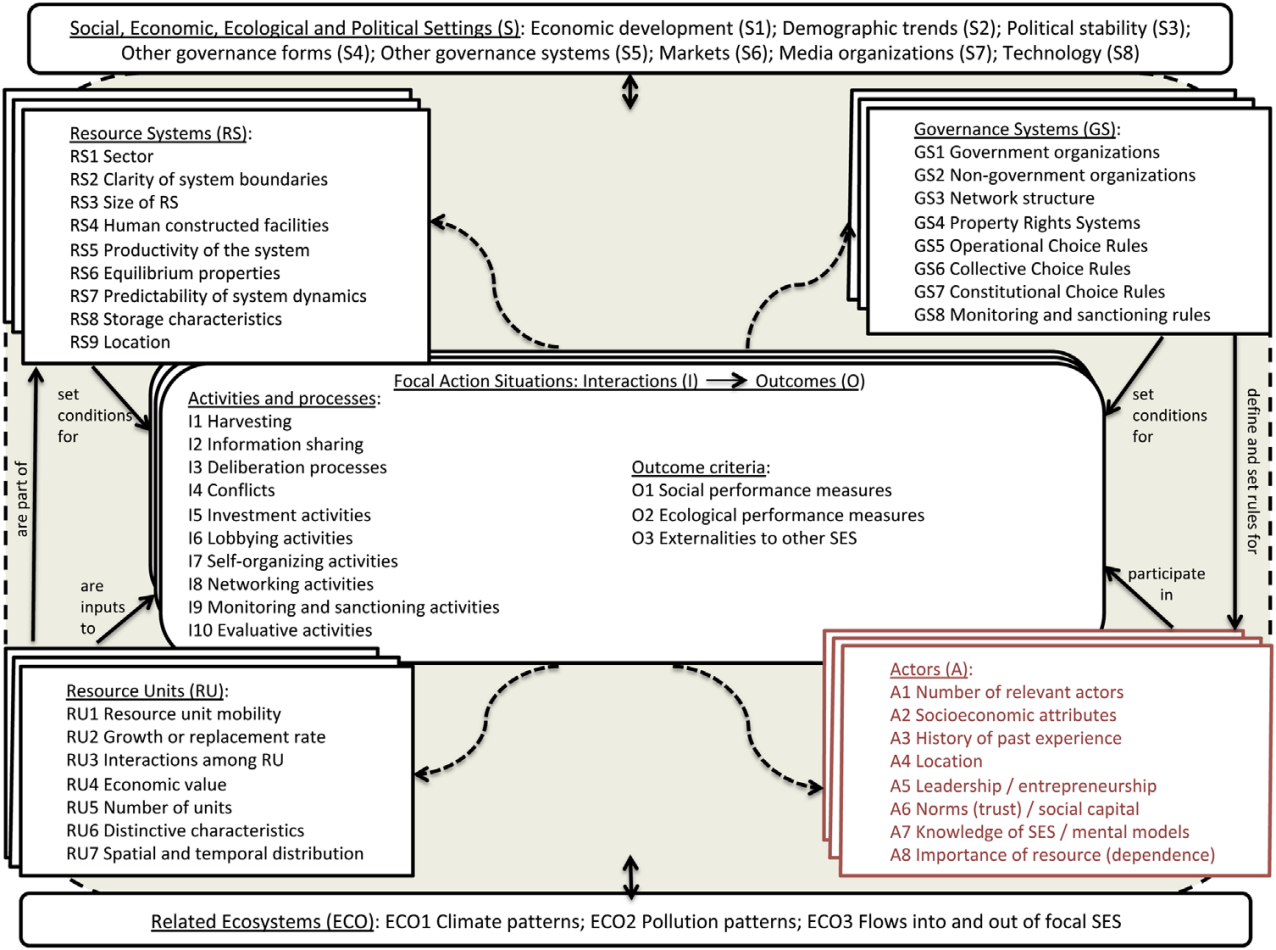
Social-ecological framework (as updated in [9] with the most recent set of variables included into the SESF [10].

Environment and society interactions have been closely examined in Ostrom's work on social-ecological systems [[7], [8], [9]]. Social-ecological systems were defined as "ecological systems intricately linked with and affected by one or more social systems" [10,11]. Ostrom’s framework provides a basic vocabulary of concepts and terms and allows making cross-institutional comparisons and evaluations [9]. Fig. 1 describes the different subsystems (resource units and systems, governance and users) interacting together within a given SES [7]. The box *actors* is in red because this paper focuses on the interactions between the A4D indicators and the *actors*’ variables.

Ostrom’s framework aims at understanding complex interactions within different systems and at various scales [12,13]. The framework is composed of 8 first-level core sub-systems defining the interactions of the SES: *Social, Economic and Political setting* (S) and *related ecosystems* (ECO) will interact with the 4 multi-linked subsystems (or tiers) that are: *resource systems* (RS), *resource units* (RU), *governance systems* (GS) and the *actors* (A). Ostrom proposes a set of second-level variables (second-tiers) to express the main features of each subsystem (Fig. 1; definitions in Annex 1) and leaves researchers the choice to add second or even third-level variables in function of their analysis [7]. These second-level variables influence sub-systems' inputs on the system. For instance, *size, productivity* and *predictability* (of the *resource system*) will influence *resource system's interactions* and *outcomes* in the SES. *Mobility* will influence *resource units*, while the *actors* system will depend on their *number*, *leadership*, *norms*, *resource dependency*, *social capital* and *knowledge*. *Outcomes* achieved in a SES depend on subsystems and on their interactions.

SESF allows combining and bringing together knowledge coming from different disciplines and finding out about those SESs' sustainability in order to improve their governance and management. Many researchers have used the SES framework in diverse environmental governance cases study [10]. However, an array of specific methods can help informing the SES. This paper shows how the Actor in 4 dimensions (A4D) can inform many elements of the SESF.

## A4D description: origin, dimensions, specificities and assets

**Fig. 2:**
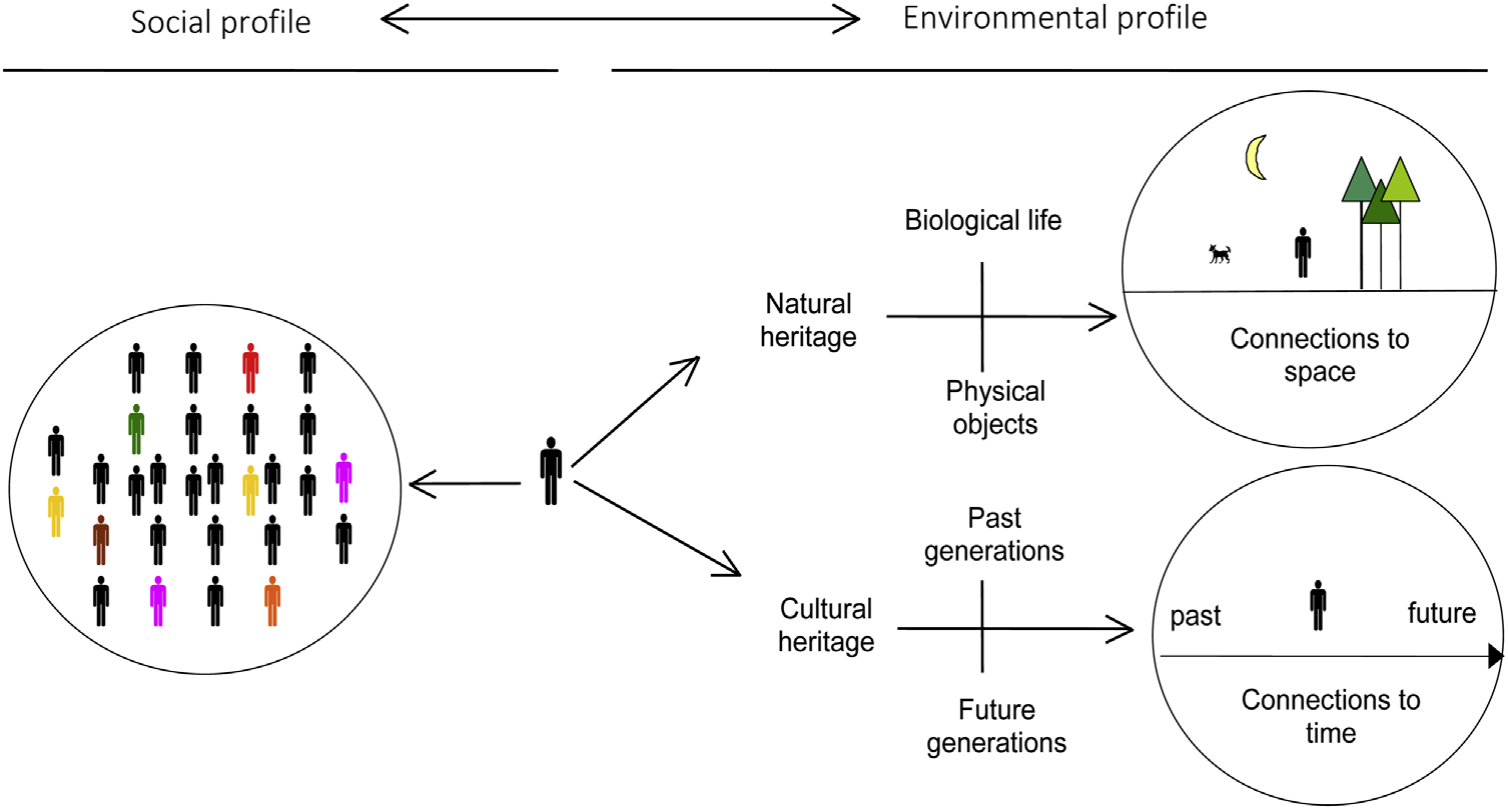
Explanations of social and environmental profiles (taken from [14]).

The A4D multidisciplinary conceptual model is an original methodology studying local environmental governance. The A4D model defines the territory as a socio-environmental system, that is to say, as the interrelations between social relations (links between actors) and environmental relations (links to nature). Therefore it aims at qualifying the relations between individuals (an actor's social profile), whether they are strong (powerful) or weak actors; and the relations between humans and non-humans (an actor's environmental profile), whether they are future generations or other species [14] (Fig. 2). It was developed both as a theoretical and as a methodological model. It aims to examine actors' perceptions on environmental and social issues, on a given space. It was designed to understand the overall stakeholders' dynamics on a territory, based on the analysis of different practices, representations and knowledge between actors about the environment and about the actor system.

**Fig. 3:**
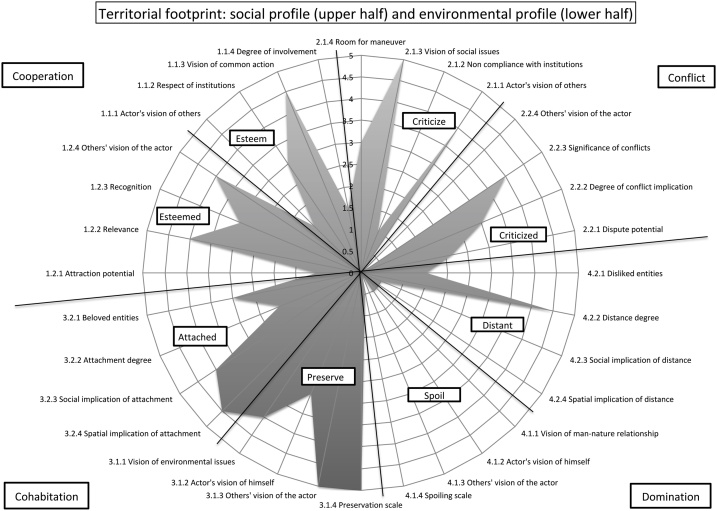
A4D territorial footprint of a local fisherman in Maio, Cape Verde.

The A4D results allow producing what their authors call the "territorial footprint", an illustration of social and environmental profiles on a territory forming a spider web graph (Fig. 3).

Each stakeholder (actor of the system) has an individual footprint, which is produced by the compilation of 32 variables (described and defined in Annex 2) comprised in eight sub-dimensions, making the model both qualitative, by the approach chosen, and quantitative, by the results analysis chosen. The four main dimensions of the model are: *cooperation* and *conflict* (social profile); and *cohabitation* and *domination* (environmental profile). The 8 sub-dimensions of the model (*esteem; esteemed; criticize; criticized; attached; preserve; distant; spoil*) inform about direct and indirect positioning of an actor within the SES, each footprint being informed by the actor discourse about himself (one of the assets of the A4D model is this reflexivity) as well as the others discourses about the actor. The A4D investigates within its social profile if an actor criticizes/esteems others and in return, if he is esteemed/criticized by others. Following the same logic with the environmental profile and based on the idea that actors influence the territory as much as the territory influences them, we analyze if an actor appears attached/distant to his environment, and if he appears to preserve/spoil natural assets. The 32 variables give different information on the actors' tendencies within those dimensions through the analysis of their practices, representations and knowledge. The footprint, illustrating stakeholders' involvement and interactions, allows one to analyze common and divergent points between individuals and groups (if multiple individual footprints are grouped by making an average of actors’ indicators’ notation, detailed in Annex 4). Footprints comparison underlines common values, which can be useful to work towards new environmental governances.

In order to study actors' positions in their SES, the A4D model is based on 1–2 h long semi-structured interviews that ask actors specific and broader questions on their environment and its governance. In this specific case (the study of Maio’s marine governance), 43 actors from various backgrounds and sectors have been interviewed. The interview guide is composed of 5 sections: structural data; ecological assets; social assets; personal actions and prospective (Annex 3 presents an example of an interview guide for fishermen). Each interview is decrypted through a discourse analysis (qualitative approach) and informs the territorial footprint (quantitative approach).

For instance, questions related to the social realm, in the dimension *conflict* and sub-dimension *criticizes* would be: "whom most deteriorates the environment; who are your enemies/opponents?". Depending on the actors' answers, the *vision of others* component would score low (0: the actor doesn't talk about others, or only talks positively about them) to high (5: the actor stands against some other actors' projects and clearly identifies its enemies/opponents). The result would then be reported on the spider web, in front of the *vision of others* component (Annex 4 shows how answers can be translated into indicators' scores). Fig. 3 shows a fisherman's footprint (hand line fishing). It appears that its left side (*cooperation* and *cohabitation*) is wider than its right side (*conflict* and *domination*). This actor especially preserves (via a simple and non-invasive lifestyle) and is attached (even if he sees some issues, limiting its attachment) to his environment (environmental profile). His social profile shows that he is esteemed (for his traditional and vital role in Maio's community) but that he is involved in important social issues (in this case, mostly with industrial fishermen).

The A4D can reveal important information about the actors' interactions and has the potential to be used by governance agents and actors themselves, by improving the dialogue on environmental issues. Managers could use this information and the whole footprint to better understand communities and actors' dynamics (all questions and related scores for the indicators are detailed in Annex 3 and 4). If discussed in a participatory governance process, it could help to target where mediation efforts need to be concentrated, what underlying elements explain the actors' perceptions, and where different interests need to be thoroughly discussed and negotiated. The A4D and its resulting territorial footprint thus can be a vehicle of data and debate, representing an operational tool for a mediator, and transferable to multiple territories and resources [27].

## Method validation (data that validate the method and connect the SES and A4D frameworks)

In order to parallel the A4D with Ostrom's framework, this part is dedicated to specifying links between both approaches, and Annexes 1 & 2 detail those indicators with definitions. We focus here on the components of the actors' category of the SESF, where the analysis of perceptions takes an important place. This subsystem notably reflects how interviewees see themselves as well as other actors in relation with the environment (i.e., A4D's social and environmental profile). The actors' subsystem of the SESF thus presents many common elements with the A4D's indicators. Information was taken from [7,8,12,15,16] for the SESF, and from [17] for the A4D. Previous research underlined the need for better and uniform definitions of the SESF tiers, as well as for methods to measure them [10]. SESF actor’s sub-tiers’ linkages with the A4D variables are described below.

**Fig. 4:**
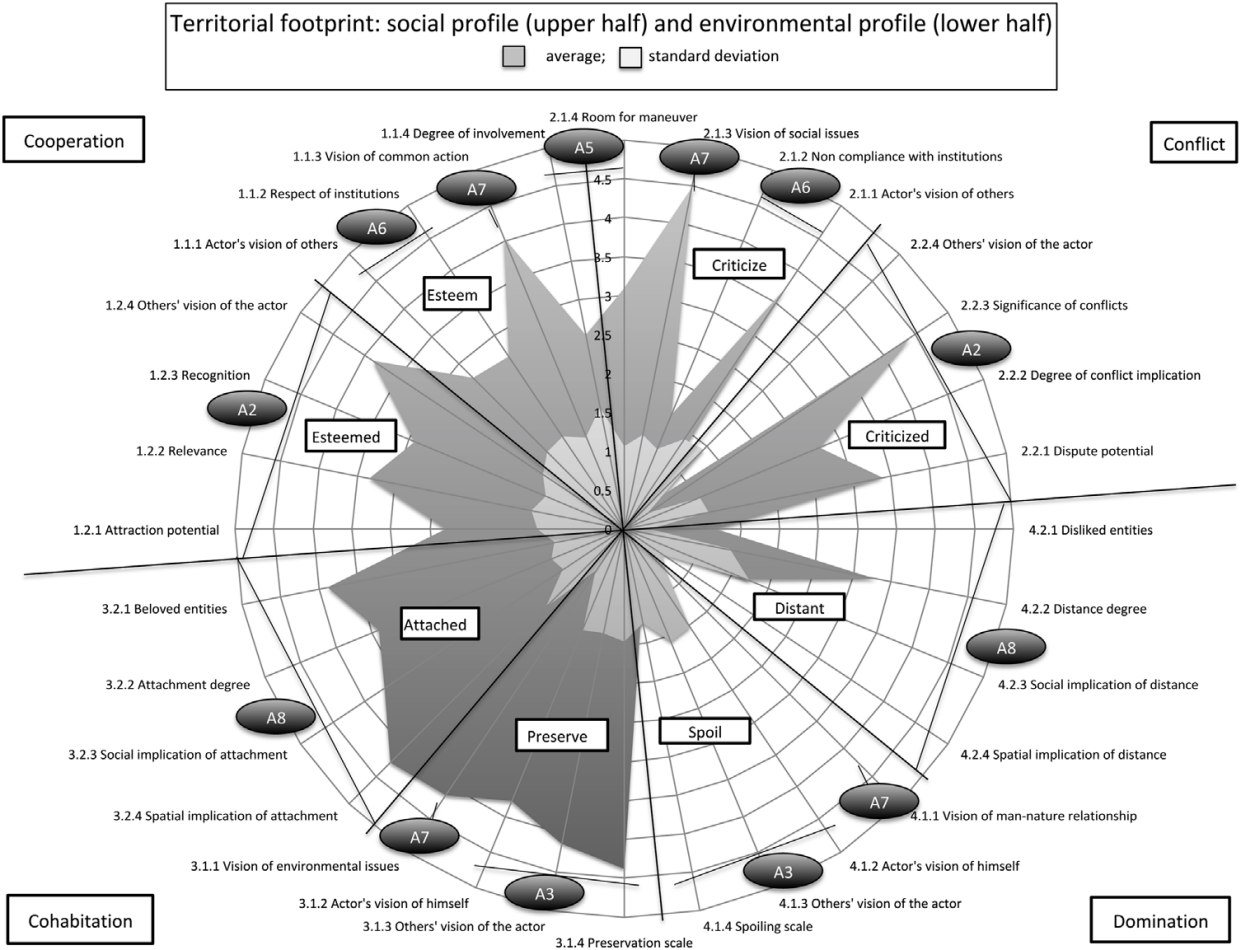
Fishermen’s group territorial footprint (1.1.1–4.2.4), with equivalent SESF Actors sub-tiers’ indicators (A1–A8).

The A4D model has been tested on various territories with very different environmental issues (ex: water management on the Kilimandjaro [14]; fisheries governance in Cape Verde [18,19]; gravels management near the Loire River and wetlands in the Basque country [20]). We chose to exemplify the parallel between the SESF and the A4D by showing the results of our analysis of governance systems of a small-scale fishery on Maio island (Cape Verde)^2^, which are detailed in the paper “An analysis of actors’ perceptions of Maio island’s (Cape Verde) marine governance” in the journal Marine Policy [19]. Fig. 4 shows the fishermen's group footprint, with A4D indicators related to *Actor's* sub-tier of Ostrom's SESF. Each sub-tier can be found on the footprint (A2 to A8) and connections between both frameworks are explained hereafter.

## Actors (A) Tier [7,8,16]

### A1 Number of actors

The A1 sub-tier is defined as the number of actors affecting decision-making processes in the SES.

The aim of the A4D model is to meet and interview all actors, or their representatives, concerned by environmental issues on an area in order to analyze the stakeholders’ dynamics. Therefore, the A4D model does not include a specific indicator concerning the number of actors, which is context dependent but identifies *strong* and *weak* actors, as well as *absent* actors (such as future generations and other species) [20], making the *Actor* category of the A4D more inclusive than the SESF *a priori*. Even if the number of actors may inform the sample size, and thus involve a relative representativeness, it does not inform the actors' degree of power or influence. Effectively, an actor may have an important influence on other actors or on specific resources, making its general input on the SES more important than another with less assets. The A4D interview's method, with snowball references for other actors to interview, allows one to target all stakeholders of an area, and thus collect the diversity of actors' perceptions. It also identifies power relations of different individuals and groups.

In the case of the small-scale fishing community of Maio island, the total community comprises between 5000 and 7000 people, with officially 137 fishermen and 89 fish saleswomen [21]. We considered different groups of actors which could be integrated in the SESF: small-scale fishermen, fish saleswomen, regional, national and municipal decision-makers and managers, other economic actors, academics, and finally, unaffiliated individuals. In total, we conducted 43 interviews (of which, 12 artisanal fishermen and 2 fish saleswomen).

### A2 Socioeconomic attributes (economic and cultural)

The A2 sub-tier is defined as social and economic characteristics of actors that affect the activity's dynamics.

In the A4D, actors' features are examined as having a potential for cooperation, or on the opposite, a potential for conflict with other actors. They are illustrated through the two main social sub-dimensions *esteemed* and *criticized*. The sub-dimension *esteemed* comprises the indicators *attraction potential*, *relevance*, *recognition* and *others' vision of the actor*. These indicators relate to the extent to which an actor's assets are numerous, un-ordinary, needed and appreciated by other actors. The sub-dimension *criticized* includes the indicators *dispute potential*, *degree of conflict implication*, *significance of conflicts* and *others' vision of the actor*. These indicators are directly opposed to the *esteemed* indicators. They relate to the extent to which an actor's shortcomings are numerous and generate conflicts, the importance of the conflicts they create and the impacts that those shortcomings have on other actors.

In Maio's small-scale fishermen group, socio-economic attributes generate more cooperation than conflicts (the sub-dimension *esteemed* is greater than the sub-dimension *criticized*) despite the importance of conflicts with industrial fisheries for declining fish stocks, and the pervasiveness of this issue for the whole socio-ecological system (indicator *significance of conflicts* rates high: 4,5/5).

### A3 History of use/of past experience

This A3 sub-tier is defined as past interactions that affect current actor's behavior and activity's dynamics: crises, duration.

Referring to the A4D, history and past experiences explaining an actor's vision on resources use is illustrated by the sub-dimensions *preserves* and *spoils* in the environmental profile. These sub-dimensions also include other stakeholders' vision on the actor's experiences with resources use. The *preserves* sub-dimension is composed of the indicators: *actor's vision of himself*, *others' vision of the actor* and *preservation scale*. They relate to the actor's vision of his own environmental behavior, to the people recognizing his preservation qualities and to the extent of his effective environmental protection. The *spoils* sub-dimension is composed of the indicators: *vision of himself*, *others' vision* and *spoiling scale*. They refer to the actor's feelings towards past, present and future practices; and to the ways other actors perceive his (more or less important) environmental-spoiling activities.

In Maio's small-scale fishers' case, the whole *preserves* sub-dimension scores way higher (4.5/5) than the *spoils* sub-dimension (1.5/5). Fishermen thus consider their impact on the socio-ecosystem as being positive, which means that they consider that they mostly preserve the environment (and have always done so), and that environmental issues are not related to their group's actions. Others also see them as preserving (much more than spoiling) agents.

### A4 Location

Location is defined as the physical place where actors are in relation to the resource itself and to the market.

Within the A4D model, the territory's location and description is scoped in a first observatory and field-familiarization phase, before proceeding to the interviews. This first territorial diagnosis aims at identifying the different groups present on the area. Then, by collecting the diverse perceptions of the territory, the A4D helps to delineate the territory's boundaries as perceived by the actors. Furthermore, the sub-dimensions on place attachment and place distance explore the attraction and repulsion of specific objects of the territory, and thus inform on the liked and disliked physical objects of the territory, precisely located.

Maio island being very isolated from Santiago (Cape Verde's capital) and other islands due to a great lack of appropriate transportation between islands, and because of its small size (275 km^2^), the island and its (vital) surrounding waters are the most appropriate scale to study SES.

### A5 Leadership

Leadership is defined as a useful skill to organize collective action (people tend to follow actors with good leadership).

In the A4D, leadership is illustrated by the sub-dimensions *esteem* and *criticize*, and especially by the indicators *degree of involvement*, which concerns the extent to which one actor respects the system's norms and values (passively or pro-actively, with more leadership); and the indicator *room for maneuver* that reflects the actor's capacity to mobilize his assets to negotiate with other actors. Together, they inform on the actor's potential to organize collective action.

For local fishermen, the *degree of involvement* as well as the *room for maneuver* indicators score relatively low (respectively 2.5 and 3/5), reflecting a quite poor leadership. In fact, as every fisherman does his own business and survives as such, without important collaboration effort, they appear to be satisfied with their low influence on others. Even if they don't respect (recognize and value) the actual system and institutions, fishermen are generally poorly motivated by the idea of more involvement to change this imperfect but still functional system.

### A6 Norms and social capital

Norms and social capital are defined as the degree by which individuals can draw upon or rely on others for support or assistance in times of need: in brief, it represents their trust in others.

In the A4D, indicators of the *esteem* and *criticize* sub-dimensions are relevant to inform on the degree of trust of the actors, especially the indicators *actor's vision of others* and *respect of institutions* on the cooperation side, and *actor's vision of others* and *non-compliance with institutions* on the conflict side. Those indicators respectively refer to the actor's positive perception about others and their projects (thus including trust and support); to the actor's compliance and mobilization in conformity with state and institutions' norms and programs; to the actor's critiques of others and their projects (i.e. distrust and dissidence); and finally, to the actor's disagreement degree on institutions and programs (which is often linked with distrust and lassitude toward unsuccessful initiatives).

Maio's fishermen showed little respect for institutions and others. Even if they don't formally disagree with institutions, they feel an important lassitude and exasperation towards their ineffectiveness. They also criticize other actors, industrial fishermen first, but also corrupted or lazy managers. They consider that local police, managers and responsibility-tasks employees don't thoroughly do their jobs. However, they explain this attitude by the small size, small population and low technical, human and financial means of the island, which prevents people from properly enforcing laws and positive initiatives.

### A7 Knowledge of SES and mental models

Knowledge of SES is defined as the degree to which stakeholders understand and make sense of the characteristics and/or dynamics of the SES.

Apprehending convergences and divergences of knowledge among actors is a great part of the A4D model, therefore it is expressed through various indicators. On the social profile, *vision of common action* (*cooperation*) and *vision of social issues* (*conflicts*) inform about the type of knowledge mobilized by the actor on social issues. On the environmental profile, *vision of environmental issues* (*cohabitation*) and *vision of man-nature relationship* (*domination*) inform about the type of knowledge mobilized by the actor on environmental issues. These indicators respectively relate to an actor's understanding of his social environment; to his perception of antagonist actors and issues; to his awareness and feelings towards environmental issues; and finally, to his perception of actors and issues that are detrimental for the environment.

Maiense's fishermen consider that there are important social and environmental issues in the SES, the most important one being foreign and industrial fisheries, that increasingly deplete the stocks they used to feed on (e.g. tunas) and come always closer to (and even enter sometimes) their restricted local fishing zone (3 nautical miles from shore). They consider fishing agreements in Cape Verde's waters, boat motorization and the arrival of scuba diving tanks as past events that increased the SES vulnerability. In future years, some feel apprehension of mass-tourism while others wish it could enrich their island.

Fishermen present specific and detailed knowledge on the species they fish or used to fish, on social roles of other actors, on certain groups' corruption, etc. However, Maio's community doesn't like to dig too far in their peers' lives, making them unaware of some problems or preventing them from acting to improve the SES.

Fishermen see their own action as positive for the SES (the *Vision of environmental issues* indicator rates 4/5 and the *Vision of man-nature relationship* indicator rates 0.5/5), which reflects their awareness on the SES, and their attitude of preservation and low domination on the socio-ecosystem.

### A8 Importance of resource/resources dependence

This element is defined as the source of monetary income, livelihoods, cultural values, practices and services coming from the resources to the benefit of the actors.

In the A4D, all indicators of place attachment and detachment (*attached* and *distant* sub-dimensions) directly inform this variable. Indeed, these 8 indicators give information on what types of territorial entities are liked or disliked, how the bounding or detachment can be characterized, and what the social and spatial implications of these different affective links are.

In fishermen's case, *beloved or highly valued entities* (tunas, groupers, mackerels, etc. as well as their boats and the sea itself) rate quite high (4/5) while the *degree of bounding* is slightly lower (3.5/5), because many expressed the intention to quit fishing. This is due to declining stocks, to harsher fishing conditions as they need to go further at sea with their small embarkations, and to changing climate and sea conditions, making their work increasingly unsafe and unpredictable. *Social implication of attachment* (3.2.3) is also quite high (4/5), explained by the fact that a degradation of fishermen entity (fishing stocks; fishing capacities, etc.) makes some of them wish to quit fishing, while others still want to continue their life vocation. *Spatial implication of attachment* (3.2.4) is also high (4.25/5) because fishermen are greatly aware that a decline of their entities means a threat for the whole SES. They also would like to transmit to future generations the pristine and rich sea that they used to know, as well as traditional activities like line fishing. In summary, fishermen are attached to their SES, but they strongly feel the necessity to distance from it because of their beloved and subsistence entities' decline.

## Nota bene

Although this paper scopes Ostrom's SESF *Actor* tier in order to parallel with the A4D model, we wish to end this part by adding that other elements of the SESF can also be informed by the A4D, such as *Governance, Interactions* and *Outcomes* subsystems. As an example, for the *Interactions* subsystem, the conflicts sub-tier constitutes a whole dimension of the A4D, being thus addressed with lots of details, as well as social bounds (ex: *Interactions'* sub-tiers on self-organizing and networking activities). Concerning the *Outcomes* subsystem, the A4D measures socio-economic and ecological performance through actors' perception of types of social and environmental bounds, and through the importance that they give on the mentioned issues. When enumerating past, present and foreseen elements that have or could disturb their well-being and the whole SES, they inevitably refer to outcomes, quantifying these outcomes within a human subjective scale.

## Additional information: introduction on SES and A4D

Social-ecological systems (SES) are increasingly used to study human-environment systems [22]. The Social-Ecological Systems framework (SESF) was created by Elinor Ostrom to better understand them [7,16]. It received much attention since 2007 [10]. However, even if the SESF is an important analytical framework [12,23], its operationalization remains elusive [12,24]. Also, even if stakeholders' participation is promoted by many authors [2,6,25] and even if Ostrom encourages researchers to build their own frameworks in collaboration with the system stakeholders, the SESF has been criticized for its difficulty in showing and explaining diverse actors' viewpoints, perceptions or intentions [2,4,5], which influence SES interactions and outcomes.

Sébastien and Paran [28] developed the Actor in 4 dimensions methodology (A4D) in 2006 [17] in order to analyze and model an actor's relationship with other actors (social profile), and his relationship with nature (environmental profile) [14]. In this regard, the A4D model informs specifically the *Actors*' sub-system of the SESF, even if it can also give useful insights for other framework's sub-systems.^3^

## Additional information: discussion on the A4D comparison with the SESF

### Individual perceptions and discourses

**Fig. 5:**
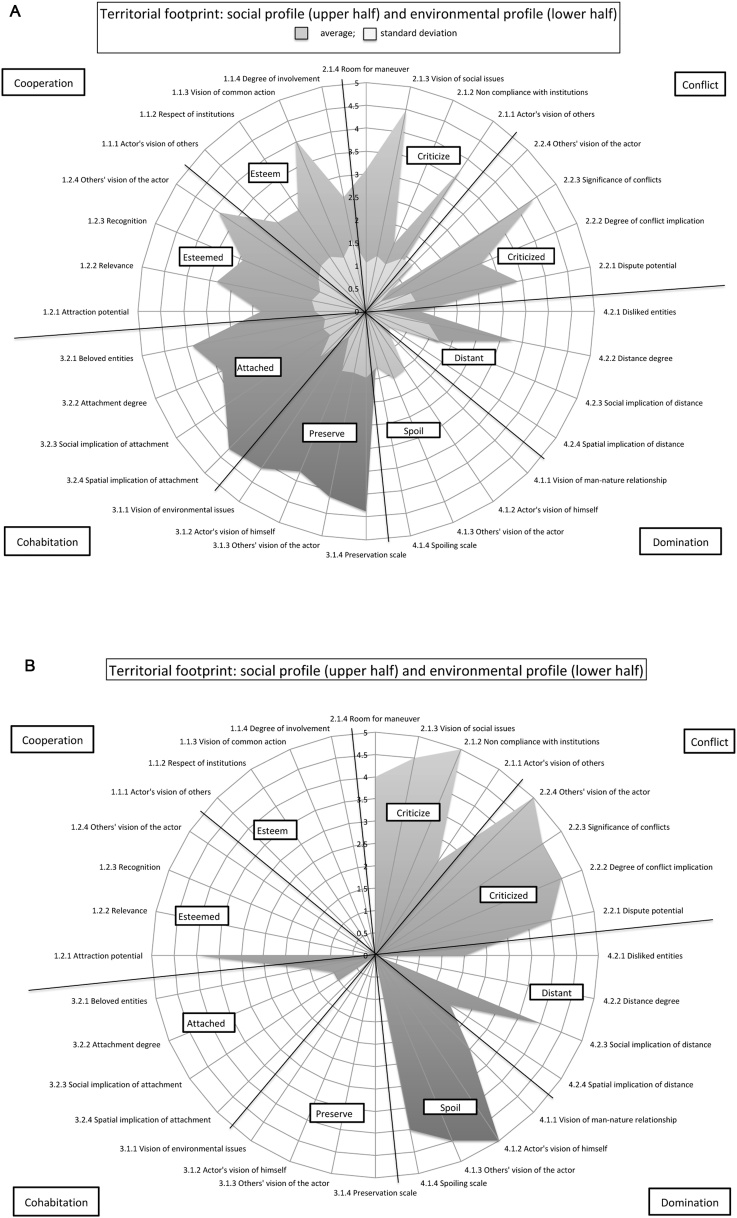
Comparison of grouped footprints (12 local fishermen to the left - A) and individual footprint (spearfisher/diver - buzio fisherman - on the right - B).

The first contribution of the A4D model towards the SESF is that of the analysis of individual perceptions. The A4D is designed to gather and study individual perceptions (including intentions), or mental models and belief systems [1,4,5,10], which influence interactions between individuals, and between individuals and their environment, with unique outcomes from one person to the other. As the SESF only analyses groups of actors, it can be blind to some individuals that would make a difference on the whole system [5]. In our case study, this is what happened with a fisherman who dives to gather buzios (gastropods) in huge quantities almost each day. Even if most other fishermen are respectful of the laws and of the environment, one single fisherman with a high degree of social conflict and environmental domination could greatly affect the SES (without being identified in the global picture of the SES; see Fig. 5).

SESF is a great schematic representation of a complex system. However, it could better incorporate individual perceptions and their impacts on the system [5]. The A4D methodology and its territorial footprints provide a tool to better understand the diversity, repartition and impacts of these perceptions.

In the same line, Thiel et al. [10] highlighted the lack of discussion on the role of discourses in 20 studies using SESF. By analyzing each actor's discourse and by underlining which elements of their vocabulary best reflect their mindset on different issues, the A4D allows researchers to use discourses to explain *Interactions* and *Outcomes* of the SES. For instance, some fishermen considered that the sea is more powerful than everything; that it can recover from any harm. They thus see fish stocks declining, but remain confident in their natural/spontaneous recovery, which is also related to their religious fervor (beliefs). This may explain an attitude of certain laxity towards national decisions that may affect their marine environment, and *in fine*, their subsistence and livelihoods. Their discourses therefore reflect personal viewpoints, on themselves (their activities and position in the SES) and on others (other's activities and place in the SES).

Another unique attribute of the A4D theory, methodology and model, is its reflexivity. The A4D illustrates others' visions and discourses on an actor as well as reflecting the actor's individual perceptions on himself. In the course of an A4D interview, actors are asked about how they see themselves in the SES: if they think that they have a beneficial or detrimental effect on the environment; if they think that other actors appreciate their work and role in the SES, etc. This does not seem to be integrated in Ostrom's framework, although people perceptions of themselves, of their capacity to change the SES, etc. are essential elements affecting their real action on the SES [4,5]. The A4D then informs the *Actor*'s sub-system and second-tiers and goes further into the actors' analysis through this reflexivity.

### Power relations and global governance

Of the 20 case studies analyzed by Thiel et al. [10], none or few discussed the role of power dissymmetries in explaining SES. This limitation may be due to these specific case studies or to the framework itself. Even if not central to the SESF, conflict and power relations analysis is partly integrated in the "rules in use" of the SESF by showing which group or individual's interests prevail effectively in the SES, especially when compared with formal statements [26,27]. Still, power relations are under-represented in the SESF [27,28].

As power relations are often linked to individuals' assets (goods, contacts, job positions, or other elements such as blackmail), it is easier to include power stakes into discussion when analyzing individuals and their perceptions in the SES, instead of groups. Power relations are at the heart of the A4D, which aims at analyzing which actors and elements influence the most environmental issues and governance. For instance, indicators of the "esteemed" and "criticized" sub-dimensions refer to power relations based on money, on knowledge, on assets uniqueness and on social position and influences. Regarding the environmental profile, power relations appear mainly in the ability of actors to protect or destroy environmental assets on the territory.

By classifying actors as *strong*, *weak* and *absent* actors within a SES, the A4D deals with unequal power relations [29]. While *strong actors* will generally be reflected in effective decisions and actions on the SES, *weak actors* are those who do not have the best assets in the negotiation (charisma, power, relations…) to impose their choice, their moral value and defend their interests. These are underrepresented contemporary humans who can bring unthought-of elements to the territorial debate, such as vernacular knowledge, a memory of the place, territorial ties [29]. *Absent actors* represent biological living and future generations and are embodied by non-human actors and non-contemporary actors, those who cannot be present at the negotiating table and who are nevertheless stakeholders. While it is important to find a negotiated solution to conflicts between contemporary humans over the uses of a common good, it should not be adopted to the detriment of absent actors [30]. The A4D, by identifying spokespersons speaking in the name of *weak* and *absent* actors (reflecting their interests), allows their voice to be heard, or to be integrated into the negotiation process. All actors (*strong, weak* and *absent* actors) need to be integrated into the SES analysis, because analysis based solely on strong actors' perceptions could lead to environmental degradation [29]. If all put together, or if grouped without careful attention, *strong, weak* and *absent* actors could all be mixed, thus not properly reflecting underlying power relations. This - dissociation of power relations in place - feature of the A4D, could then help to "reconnect" SES studies and decision-relevant information that policymakers could use [31].

In Maio, a group that appeared to be a good spokesperson for future generations as well as for marine life was fish saleswomen. Effectively, this group was very attached to the environment and to its entity (fish, for subsistence and traditional meals, but also species that had disappeared or were threatened, such as some shark and ray species). They were very concerned by Maienses future generations' ability to continue fishing and pursue their traditional livelihood activities. For that reason, they wanted to be heard in order to preserve Maio's SES. Their views on common action (participative governance) were the highest of all groups of actors. Therefore, fish saleswomen, willing to be involved in decision-making and attached to their entities (job and fish, for themselves and foremost, for future generations), are a good example of spokesperson for *weak* and *absent* actors in the A4D.

Governance systems are guided by individuals, each having their own perceptions on social and environmental stakes, and thus influencing decision-making as well as related processes (governance communication, information and effective enforcement). Individual perceptions influence collective access to natural resources, to their property and usage rights. In Maio's case, some decision-makers think that fish overexploitation is mainly caused by local illegal practices and therefore regulated those activities. Although these need to be limited, fishing pressure from foreign fisheries is a greater threat, on most interviewees’ point of view. Therefore, decisions do not overcome the main cause of resources overexploitation. This dichotomy between perceptions (or intentions [5]) and actions can only be addressed by the SESF if it corresponds to a group's mental model, which appears more clearly in the A4D by a comparison between individuals and groups' footprints and therefore, by a comparison of their environmental and social profiles.

Using territorial footprints also allows participative governance and information sharing between actors, an essential criteria for good co-management [32]. The A4D model can effectively help engaging dialogue among actors, by revealing individual intentions and perceptions, by allowing a debate about actor’s values, and by proposing a negotiation between *strong, weak* and *absent* actors.

## Conclusion

We showed that the A4D can be a useful method to complete and operationalize the SES framework, specifically its *Actor’*s subsystem, by incorporating individual strategic (subjective) elements in a systemic framework and by adding a reflexive perspective. Furthermore, the association of A4D model and SES framework contributes to a reflection on governance issues, and on the necessary integration of individual perceptions and power relations in the analysis of local governance systems. In that perspective, the A4D and its territorial footprint can be powerful to go further in the SES analysis, and in its diffusion towards local actors.

## Footnotes

1 A tier is a logical category that contains subdivisions or lower-level tiers, called sub-tiers. For example, *Resource Systems* (RS) represents a top-tier category with second-tier subdivisions denoting such characteristics as its *size* (RS3), *resource sector* (RS1), etc. (Fig. 1).

2 fieldwork from two on-site missions: summer 2013 and autumn 2015

3 The A4D having been developed by French researchers, and having mainly been spread in French-written journals and research networks, this paper presents a great occasion to position the A4D methodology within a famous English-speaking world framework studying environmental governance: the SES framework.
