# Supplementary material for: The Actor in 4 dimensions: A relevant methodology to analyze local environmental governance and inform Ostrom’s social-ecological systems framework
Source: MethodsX. 2019 Jul 31;6:1798–811. doi: 10.1016/j.mex.2019.07.025 (PMC6700404; doi:10.1016/j.mex.2019.07.025)
Supplement: Supplementary file 1 [file mmc1.docx]

Annex 1: First and second-tiers of the SESF

(Ostrom 2009, McGinnis and Ostrom 2014 , Delgado-Serrano and Ramos 2015, Thiel *et al.* 2015)

Resource System (RS)

RS1 Sector (characteristics of a RS distinguishing it from other RS)

RS2 Clarity of system boundaries (biophysical characteristics making it feasible for actors to determine the RS start and end)

RS3 Size of resource system (spatial extent; includes carrying capacity)

RS4 Human constructed facilities (anthropogenic structures facilitating resource management)

RS5 Productivity of the system (generation rate of biomass units determined by production-consumption rates per unit of time, surface or volume: includes stock status and biophysical factors)

RS6 Equilibrium properties (Influences - positive and negative - on the equilibrium of the RS: interaction between species, or between biological and anthropological systems)

RS7 Predictability of system dynamics (degree to which actors can forecast or identify patterns in environmentally driven variability)

RS8 Storage characteristics (degree to which the resource units can be held captive until harvested: in natural habitat / in human-designed facility)

RS9 Location (spatial and temporal extent where resource units are found by actors)

Resource Units (RU)

RU1 Resource unit mobility (sessile or moving)

RU2 Growth or replacement rate (absolute or relative descriptions of changes in quantities (x) of RU over time (t)

RU3 Interaction among resource units (ex: during different life stages affecting the future structure of the population)

RU4 Economic value (value of the resource units in relation to the portfolio of resources available to actors / market or non-market)

RU5 Number of units (number / amount of resources units harvested or that could be potentially harvested)

RU6 Distinctive characteristics (markings or behavioral patterns in RU, which affect actors' behavior toward them)

RU7 Spatial and temporal distribution (availability of the resource in space and time)

Governance System (GS)

GS1 Government organizations (presence or absence of different organizations at local level)

GS2 Non government organizations (presence of NGOs)

GS3 Network structure (network configuration at local level and their interactions)

GS4 Property rights system (local property-rights systems and their relation to resource management)

GS5 Operational choice rules (local rules for defining Who, How, Where, When and Why have access to local natural resources)

GS6 Collective choice rules (formal recognition of property / use rights; traditional use privileges; operational rules)

GS7 Constitutional choice rules (legal framework defined by regional and national governments)

GS8 Monitoring and sanctioning rules and processes (locally adapted processes to monitor and sanction natural resource use and management strategies)

Actors (A)

A1 Number of actors (affecting decision-making processes in the SES)

A2 Socioeconomic attributes (characteristics of actors related to social and economic dimensions affecting the activity's dynamics)

A3 History of past experience (past interactions that affect current actor's behavior and activity's dynamics: crises, duration)

A4 Location (physical place where the actors are in relation to the resource itself and the market)

A5 Leadership (actors who have skills useful to organize collective action and are followed by their peers)

A6 Norms (trust) / social capital (degree by which individual(s) can draw upon or rely on others for support or assistance in times of need: trust in others)

A7 Knowledge of SES/mental models (degree to which stakeholders understand and make sense of the characteristics and / or dynamics of the SES)

A8 Importance of resource (source of monetary income, livelihoods, cultural values, practices and services)

Interactions (I)

I1 Harvesting levels (quantity of resource harvested by different users)

I2 Information sharing (methods for information sharing among users)

I3 Deliberation processes (deliberation processes used among users)

I4 Conflicts (existing conflicts among users)

I5 Investment activities (investment for improving and managing the resources: investor, amount and destination of investment)

I6 Lobbying activities (internal and external influence capacity of the users)

I7 Self-organizing activities (internal rules for the extraction and management of resources among users)

I8 Networking activities (Networking and partnership activities of the users within and outside the community)

I9 Monitoring and sanctioning activities (on the use and management of the resources and their performance)

I10 Evaluation activities (processes of evaluation of the resource situation and of the effects of management initiatives)

Outcomes (O)

O1 Socio-economic performance measures (evolution and impacts of the socio-economic concepts included)

O2 Ecological performance measures (evolution and impacts of the ecological concepts included)

O3 Externalities to other SES (non desired effects - positive and negative - that occur as results of the processes)

Social, ecological, political settings (S)

S1 Economic development (economic situation, including the standard of living and the economic health of the area)

S2 Demographic trends (trends, changes and status of the human population)

S3 Political stability (core regulatory framework for the country or region and regularity in the rules and values of the democratic processes)

S4 Other governance forms

S5 Other governance systems

S6 Market incentives (markets for natural resources and conservation incentives)

S7 Media organizations (number, diversity and freedom of private and public media)

Related ecosystems (ECO)

ECO1 Climate patterns

ECO2 Pollution patterns

ECO3 Flows into and out of focal SES

Annex 2: Structure of the A4D model (dimensions, sub-dimensions and indicators)

(Sébastien 2006):

1. Cooperation dimension:

1.1. Esteem sub-dimension:

Indicators

1.1.1 Actor's vision of others (the actor's viewpoint on other actors and their projects)

1.1.2 Respect of institutions (mobilization of actors' assets in conformity with norms organizing collective action, feelings towards state and institution programs)

1.1.3 Vision of common action (identification of actual or potential partners by the actor with whom he shares common objectives)

1.1.4 Degree of involvement (ways to respect the system's norms and values: from passive conformity to zealous cooperation)

1.2. Esteemed sub-dimension:

Indicators

1.2.1 Attraction potential (resources, assets and means that the actor possesses)

1.2.2 Relevance (uniqueness and availability of the actor's assets for others)

1.2.3 Recognition (assets acknowledged and required by others)

1.2.4 Others' vision of the actor (other actors' point of view on the actor and his projects)

2. Conflict dimension:

2.1 Criticize sub-dimension:

Indicators

2.1.1 Actor's vision of others (the actor's point of view regarding other actors and their projects)

2.1.2 Non compliance with institutions (actor's disagreement degree and ways to express it)

2.1.3 Vision of social issues (perception of antagonist actors and issues)

2.1.4 Room for maneuver (the actor's capacity to use his assets to be heard and negotiate with other actors)

2.2. Criticized sub-dimension:

Indicators

2.2.1 Dispute potential (elements of an actor that can be criticized)

2.2.2 Degree of conflict implication (number and type of conflicts in which the actor is implicated)

2.2.3 Significance of conflicts (importance of criticized elements and their impacts for other actors)

2.2.4 Others' vision of the actor (the others' point of view regarding the actor and his projects)

3. Cohabitation dimension:

3.1 Preserve sub-dimension:

Indicators

3.1.1 Vision of environmental issues (awareness and feelings about global and territorial environmental issues)

3.1.2 Actor's vision of himself (measures how the actor perceives his past, present and future actions of environmental protection)

3.1.3 Others' vision of the actor (measures how the other actors judge the actor for his protection practices)

3.1.4 Preservation scale (measures the type of objects protected by the actor, with time and space scales)

3.2 Attached sub-dimension:

Indicators

3.2.1 Beloved entities (the number of entities that the actor likes, their rarity, entity characterization)

3.2.2 Attachment degree (discourse on entities; bounds, values, risks associated)

3.2.3 Social implication of attachment (type of social actions put in place in front of entity degradation; type of attachment transmission)

3.2.4 Spatial implication of attachment (type of territorial actions put in place in front of entity degradation; importance of the entity in the ecosystems; skills and knowledge developed)

4.Domination dimension:

4.1 Spoil sub-dimension:

Indicators

4.1.1 Vision of man-nature relationship (values that the actor associates with nature; the perceived impact of human activities on nature and vice versa; and actors responsible of nature degradation)

4.1.2 Actor’s vision of himself (measures how the actor perceives his past, present and future actions of environmental degradation)

4.1.3 Others' vision of the actor (measures how the other actors judge the actor for his degradation practices)

4.1.4 Spoiling scale (measures the type of objects spoiled by the actor, with time and space scales)

4.2 Distant sub-dimension:

Indicators

4.2.1 Disliked entities (the number of entities that the actor dislikes, their rarity, entity characterization)

4.2.2 Distance degree (spontaneity, vocabulary, effects and reactions toward disliked entities)

4.2.3 Social implication of distance (type of social actions put in place in front of entity preservation; type of distance transmission)

4.2.4 Spatial implication of distance (type of territorial actions put in place in front of entity preservation; importance of the entity in the ecosystems; skills and knowledge developed)

Annex 3: Interview guide for fishermen

City name, date:

Introduction

Interviewer presentation / Interviewee's presentation (briefly):

Structural data:

Name:

Occupation:

Affiliated to fishermen's association? Why? If so, what role and tasks in the FA? Since?

Family? Which family members work? In fisheries?

Fishing sites?

Fishing function (main fisherman, helper)?

Fishing method (line, net, spearfishing, etc.)?

Fishing since (number of years)?

Main species caught?

Academic formation / specialization?

2. Ecological assets (marine system)

- Is the marine environment well managed?

- Is its quality sufficient?

- What does the marine environment bring to the island?

- What elements of the marine environment could be negative for the island?

- Should we protect the marine environment?

3. Social assets (stakeholders dynamics)

- Who spoils the most the marine environment?

- Who protects the most the marine environment?

- What are the conflicts on the marine environment? Why?

- Who are your opponents? Allies?

- Are you satisfied with dialogue and concertation on your island? Would you like to decide?

- Do you think that maximal community participation to decision-making is important?

- Do we need consensus to manage the marine environment?

- Do you think that certain groups feel left apart from projects? If so, why?

4. Personal actions at local scale:

- Do you try to act on mentioned issues?

- What do you do to prevent marine environment's degradation?

- What is your impact on the marine environment (positive and negative)?

- Which actions could you take to ensure a better management of the marine environment?

5. Prospective and projects in Maio (MPAs, tourism, fisheries co-management)

- Which were the main changes in the marine environment since 20 years?

- What are your feelings toward lost activities?

- What elements from the past may have most influenced the present situation of the marine system?

- How do you see your island and its marine surroundings (and resources) in 10 / 50 / 100 years? Pink and black scenarios?

- Which conflicts / issues do you foresee in the future, in relation with your marine environment?

About the marine projects in Maio:

- What is your opinion about these projects?

- What would you like these projects to achieve?

- What is your most important bound with the sea?

- Do you think that MPAs are important? Why?

- What could allow better community access and involvement in these projects?

Conclusion/ Final questions:

- What did you think about this interview?

- Would you like to talk about something else?

- Can we contact you by phone to have more information?

- Do you recommend some other contacts for this study? In general and in your sector?

Annex 4: System of notation for each variable of the A4D model (Sébastien, 2006)

Component

*Cooperation dimension* – *Esteem* sub-dimension Indicators



I.1.1. Actor's vision of others

0 : The actor never talks about the others or their projects; or talks about them in a negative way (conflict dimension).

1 : The actor talks about the others, but not about their projects, in a neutral way.

2 : The actor talks about the others and about their projects, in a neutral way. He only appreciates his partners.

3 : The actor appreciates some projects but criticizes projects' promoters.

4 : The actor shows interest for some actors and projects, besides his partners.

5 : The actor is consensual, if not enthusiastic, towards numerous actors and projects.



I.1.2. Respect of institutions

0 : The actor never talks about laws or programs, or in a negative way (conflict dimension).

1 : The actor talks about laws and programs in a neutral way.

2 : The actor talks about laws and programs in a neutral way.

3 : The actor appreciates some laws and programs.

4 : The actor appreciates some laws and programs.

5 : The actor participates to programs or voluntarily reaches out to the law.

I.1.3. Vision of common action

0 : The actor does not wish for other partners and negatively criticizes the idea of concertation.

1 : The actor only wishes for more contacts with his close circle (clients, funders) to answer only his own interests.

2 : The actor wishes for more concertation if supported by a strong actor or decision-maker (himself). He asks for power.

3 : The actor wishes for more concertation if supported by a strong actor or decision-maker (someone else). He asks for recognition.

4 : The actor wishes for concertation if limited to involved actors.

5 : The actor wishes for an open to all concertation (governance, participative approach)

I.1.4. Degree of involvement

0 : The actor does not consider any step towards others.

1 : The actor is present but is distant: passive conformity.

2 : The actor meets others to explain what they do wrong: he wants to change attitudes.

3 : The actor meets others to be better known and perceives himself as unheard.

4 : The actor participates to territorial concertations to share, listen and build together.

5 : The actor organizes territorial concertations for a common vision: very cooperative and bounding actor.

Component

*Cooperation* dimension – *Esteemed* sub-dimension indicators

I.2.1. Attraction potential

0 : The actor has no knowledge (assets), neither does he possess properties, human influence, partners no financial means (5 assets)

1 : Has 1 on 5 assets

2 : Has 2 on 5 assets

3 : Has 3 on 5 assets

4 : Has 4 on 5 assets

5 : Has 5 on 5 assets



I.2.2. Relevance

0 : None of the actor's asset is mentioned in others' speech (unavailable; possessed by all)

1 : The asset is not mentioned or possessed by all

2 : The asset answers a specific need or is possessed by more than 50% of the actors.

3 : The asset answers many needs or is possessed by less than 50% of the actors.

4 : The asset is considered as vital or only possessed by the actor.

5 : The asset is considered as vital, possessed only by the actor, and offered (common project)

I.2.3. Recognition

0 : The actor is never mentioned for his assets.

1 : His assets are required by only one actor, without citing his name.

2 : His assets are required by only one actor, by citing his name.

3 : His assets are required by many actors, without citing his name.

4 : His assets are required by many actors, by citing his name.

5 : The actor is considered vital for his assets (required by all)

I.2.4. Others' vision of the actor

0 : Neither the actor nor his projects are mentioned by others, or in a negative way (conflict dimension)

1 : People talk about the actor, but not about his projects, in a neutral way.

2 : People talk about the actor and his projects in a neutral way (only appreciated by his partners)

3 : People appreciate some of the actor's projects while citicizing him negatively.

4 : People appreciate the actor and his projects.

5 : People consider that the actor is a leader.

Component

*Conflict* dimension – *Criticize* sub-dimension indicators



II.1.1. Actor's vision of others

0 : The actor never talks about the others or their projects; or in a positive way (cooperation dimension)

1 : The actors talks about the others and their projects in a neutral way.

2 : The actor criticizes in a general, negative way, without citing names nor projects.

3 : The actor criticizes in a specific, negative way, a group of actors.

4 : The actor criticizes in a virulent, negative way, and identifies opponents.

5 : The actor stands against others' projects and precisely identifies his enemies.



II.1.2. Non compliance with institutions

0 : The actor never talks about law texts nor programs, or in a positive way (cooperation dimension)

1 : The actor regrets institutions' laxity and laws poor enforcement.

2 : The actor doesn't trust programs and blames institutions.

3 : The actor stands against laws and programs.

4 : The actor stands against laws and programs; want to change the system.

5 : The actor situates himself as an outlaw.

II.1.3. Vision of social issues

0 : The actor does not identify any opponent and no territory issues.

1 : The actor mentions territory issues in a general way (without citing names).

2 : The actor mentions opponents without linking them to territory issues.

3 : The actor identifies opponents and link them to territory issues, without getting involved.

4 : The actor identifies opponents and link them to personal issues (conflicts).

5 : The actor identifies opponents and link them to territory issues where he is involved.

II.1.4. Room for maneuver

0 : The actor never expresses disagreement.

1 : The actor soflty expresses disagreement in the interview (empathy, without citing names).

2 : The actor freely expresses disagreement in the interview (gives names).

3 : The actor expresses passive opposition during meetings with other actors (sais the truth, but makes compromises).

4 : The actor expresses a clear opposition during meetings with other actors (wants to change things)

5 : The actor expresses an active opposition during meetings with other actors (systematically disagrees and blocks progress).

Component

*Conflit* dimension – *Criticized* sub-dimension indicators



II.2.1. Dispute potential

0 : 0 to 0,16 (= average between *attraction potential* component score and the *Criticize* sub-dimension: the actor has not much assets and does not criticize much others)

1 : 0,17 to 0,32

2 : 0,33 to 0,5

3 : 0,51 to 0,67

4 : 0,68 to 0,84

5 : 0,85 to 1 (the actor has many assets and criticizes others a lot).



II.2.2. Degree of conflict implication

0 : The actor is involved in no conflict.

1 : The actor is involved in a personal, use or moral value conflict (1/3 categories).

2 : The actor is involved in 2/3 conflict categories.

3 : The actor is involved in many conflicts in 2 categories.

4 : The actor is involved in 3 conflict categories.

5 : The actor is involved in many conflicts, in the 3 categories.

II.2.3. Significance of conflicts

0 : The actor is involved in no conflict mentioned by the others.

1 : The actor is involved in a conflict rarely mentioned, which is a localized / marginal issue.

2 : The actor is involved in a conflict rarely mentioned, which is a territory issue.

3 : The actor is involved in a conflict often mentioned, which is a localized issue.

4 : The actor is involved in a conflict often mentioned, which is a territory issue.

5 : The actor is involved in many conflicts, which are major issues for the SES (crystalize)

II.2.4. Others' vision of the actor

0 : Neither the actor, nor his projects, are mentioned by the others, or in a positive way (cooperation dimension)

1 : People talk about the actor and his projects in a neutral way.

2 : People criticize the actor in a negative, general way (without citing projects)

3 : People (negatively) criticize the actor for his projects (without virulence)

4 : People globally consider the actor as a drag on the territory (virulent speach)

5 : People globally judge the actor as causing problems on the territory.

Component

*Cohabitation* dimension – *Preserve* sub-dimension indicators



III.1.1. Vision of environmental issues

0 : The actor never mentions environmental issues

1 : The actor mentions environmental issues focused on his problems

2 : The actor mentions environmental issues not linked to the territory (global issues), or mentions conservation, but only for human use.

3 : The actor analyzes environmental issues linked to the territory, and identifies causes.

4 : The actor shows concerns in front of environmental issues linked to the territory, and proposes solutions.

5 : The actor proposes nature conservation and sanctuary to solve environmental issues.



III.1.2. Actor's vision of himself

0 : The actor does not protect (nature domination).

1 : The actor shows civism only (garbage sorting; laws' respect).

2 : The actor intents to preserve.

3 : The actor limits degradation and impacts.

4 : The actor protects habitats and species (manager).

5 : The actor voluntarily preserves nature.

III.1.3. Others' vision of the actor

0 : The actor is never mentioned for his preservation / conservation actions.

1 : The actor's preservation actions are considered unimportant or irrelevant.

2 : The actor is mentioned for minor preservation actions.

3 : The actor is mentioned for encouraging preservation actions (to come).

4 : The actor is mentioned for interesting preservation actions.

5 : The actor is often mentioned for essential model preservation actions.

III.1.4. Preservation scale

0 : The actor doesn't preserve.

1 : The actor's preservation actions are localized, "for himself and now".

2 : The actor's preservation actions are localized, "for us, now and tomorrow".

3 : The actor's preservation actions are for the whole territory, "for us now".

4 : The actors preserves complex entities for future generations.

5 : The actor preserves complex entities for future generations with low means.

Component

*Cohabitation* dimension – *Attached* sub-dimension indicators



III.2.1. Beloved entities

0 : The actor shows no attachment toward any specific entity.

1 : The actor shows low attachment toward a few entities.

2 : The actor shows attachment toward many entities, based on nostalgia.

3 : The actor shows important attachment toward many entities (symbol, flagship)

4 : The actor shows love, passion toward one or many entities often mentioned by others.

5 : témoigne de l'amour, de la passion envers une ou plusieurs entités rarement évoquées par les autres



III.2.2. Attachment degree

0 : The actor doesn't mention, or simply cites the entity in his speech.

1 : The actor only mentions the entity in the context of his work activity.

2: The actor talks about the entity in personal ways and appreciates its use values.

3 : The actor talks about the entity in personal ways and appreciates its leisure values.

4 : The actor is passionate and assigns an existential value to the entity.

5 : The actor is passionate, assigns an existential value to the entity and he takes risks to protect it.

III.2.3. Social implication of attachment

0 : The actor likes his entity only for himself and doesn't wisht to transmit it.

1 : The actor's love for his (rarely cited) entity makes him separate from other actors.

2 : The actor wishes to transmit his love for a rarely cited entity, only to his parents and close relatives.

3 : The actor wished to transmit his love for a rarely cited entity without affecting his relations with others (compromise, statu quo)

4 : The actor wishes to transmit his love for an entity without affecting his relations with others (compromise, statu quo)

5 : The actor organizes common actions to transmit his love for his entity to present and future generations.

III.2.4. Spatial implication of attachment

0 : The actor could destroy nature in order to transmit his entity (belonging to modern society).

1 : The actor could destroy nature to preserve a localized natural entity.

2 : The actor wishes to transmit a natural localized entity for itselfs, without considering ecological systems.

3 : The actor wishes to transmit a natural localized entity without compromising ecological systems.

4 : The actor wishes to transmit a complex natural entity without compromising ecological systems.

5 : The actor is looking for a balanced solution in order to protect nature and his entity, considered as a complex ecological system.

Component

*Domination* dimension – *Spoil* sub-dimension indicators



IV.1.1. Vision of man-nature relationship

0 : The actor considers that nature has an existential value; that men actions are irreversible and that everyone should protect nature.

1 : The actor considers that nature has a leisure value; he is aware of environmental issues but remains optimistic (solutions are possible).

2 : The actor considers that nature has a leisure value; he identifies environmental issues but stays fatalistic.

3 : The actor only considers that nature has a use value; he identifies environmental issues but says fatalistic.

4 : The actor only gives a use value to nature, and identifies few environmental issues.

5 : The actor doesn't give any value to nature; he considers that we must dominate nature in order to limit its nuisances; technique is key to our needs' satisfaction.



IV .1.2. Actor's vision of himself

0 : The actor says that he doesn't spoil nature.

1 : The actor says that his spoiling degree is low; that he has already changed his habits.

2 : The actor says that he spoils nature less than others and that he will change his habits.

3 : The actor says that he spoils as much as other actors and that he will limit his impacts.

4 : The actor says that he spoils as much as other actors, and that he will not change anything.

5 : The actor admits destroying more than others, knows that it matters but does not regret.

IV .1.3. Others' vision of the actor

0 : The actor is never mentioned by others for his spoiling activities.

1 : The actor is rarely mentioned for minor spoiling activities.

2 : The actor is often mentioned for minor spoiling activities.

3 : The actor is rarely mentioned for important spoiling activities.

4 : The actor is often mentioned for important spoiling activities.

5 : The actor is often mentioned for catastrophic spoiling activities.

IV .1.4. Spoiling scale

0 : No spoiling activity.

1 : Temporary deterioration, minor neglect (possible resilience).

2 : Limited deterioration on an isolated entity (resilience still possible).

3 : Disturbing deterioration on an isolated entity (possible long terme resilience).

4 : Irreversible deterioration on isolated entities.

5 : Ecological systems are irreversibly spoiled.

Component

*Domination* dimension – *Distant* sub-dimension indicators



IV.2.1. Disliked entities

0 : The actor does not mention territory's shortcoming.

1 : The actor mentions only few territory's shorcomings, coming from modern society.

2 : The actor mentions few shortcoming from his natural or cultural heritage.

3 : The actor mentions many shortcomings from his natural or cultural heritage, entities that other actors dislike too.

4 : The actor mentions many shortcomings from his natural and cultural heritage, entities disliked only by the actor, who criticizes them with many details.

5 : The actor mentions many shortcomings from his natural and cultural heritage, entities disliked only by the actor, who does not know them well.



IV.2.2. Distance degree

0 : The actor does not speak about the entity, on only mentions it in his speech.

1 : The actor does not talk much about the entity; he mostly mentions nostalgia (change).

2 : The actor criticizes an important, harmful entity for the territory.

3 : The actor criticizes many entities that slow his activities.

4 : The actor criticizes many entities that question his own interests.

5 : The actor speaks and argues with hatred toward the entity, which questions his survival.

IV .2.3. Social implication of distance

0 : The entity is often mentioned by other actors, leading to discussions and meetings.

1 : The actor keeps his hatred for himself and is empathetic toward those appreciating his disliked entity.

2 : The actor does not communicate his hatred, is distant and avoids showing his entity dislike.

3 : The entity is mentioned by many actors; leading to conflicts and group division.

4 : The entity is never mentioned by other actors, leading to divisions and unsolvable conflicts.

5 : Other actors never mention the entity, leading to unsolvable conflicts with many actors.

IV .2.4. Spatial implication of distance

0 : The actor only mentions shortcomings from modern society.

1 : The actor mentions shortcomings that are natural entities, localized and without harmful impact.

2 : The actor mentions shortcomings that are natural and localized entities, making him want to escape his territory.

3 : The actor mentions shortcomings that are a natural and complex entity, making him want to escape his territory.

4 : The actor mentions shortcomings that are a natural and complex entity, forcing him to modify other elements.

5 : The actor mentions shortcomings related to his ecological system, pushing him to destroy other natural elements.
